# “An Ounce of Prevention is Worth a Pound of Cure”: Proposal for a Social Prescribing Strategy for Obesity Prevention and Improvement in Health and Well-being

**DOI:** 10.2196/41280

**Published:** 2023-02-17

**Authors:** Sisitha Jayasinghe, Timothy P Holloway, Robert Soward, Kira A E Patterson, Kiran D K Ahuja, Lisa Dalton, Sandra Murray, Roger Hughes, Nuala M Byrne, Andrew P Hills

**Affiliations:** 1 School of Health Sciences College of Health and Medicine University of Tasmania Launceston Australia; 2 College of Arts, Law and Education University of Tasmania Launceston Australia; 3 School of Health Sciences Swinburne University of Technology Melbourne Australia

**Keywords:** capacity building, community development, determinants of health, health care delivery, health care management, obese, obesity, patient education, peer education, prevention, screening, service delivery, social prescribing, social prescription, weight

## Abstract

**Background:**

Social and behavioral determinants of health are increasingly recognized as central to effective person-centered intervention in clinical practice, disease management, and public health. Accordingly, social prescribing (SP) has received increased attention in recent times. The rampant global prevalence of obesity indicates that the customary, reductionistic, and disease-oriented biomedical approach to health service delivery is inadequate/ineffective at arresting the spread and mitigating the damaging consequences of the condition. There is an urgent need to shift the focus from reactive downstream disease-based treatments to more proactive, upstream, preventive action. In essence, this requires more effort to affect the paradigm shift from the traditional “biomedical approach of care” to a “biopsychosocial model” required to arrest the increasing prevalence of obesity. To this end, an SP approach, anchored in systems thinking, could be an effective means of moderating prevalence and consequences of obesity at a community level.

**Objective:**

The proposed SP intervention has the following three key objectives: (1) build a sustainable program for Circular Head based on SP, peer education, and health screening to minimize the incidence of obesity and related lifestyle diseases; (2) increase service and workforce connectivity and collaboration and initiate the introduction of new services and activities for obesity prevention and community health promotion; and (3) enhance health and well-being and minimize preventable adverse health outcomes of obesity and related lifestyle diseases through enhancement of food literacy and better nutrition, enhancement of physical literacy and habitual personal activity levels, and improvement of mental health, community connectedness, and reduction of social isolation.

**Methods:**

This paper describes a prospective SP strategy aimed at obesity prevention in Circular Head, a local government area in Northwest (NW) Tasmania. SP is a prominent strategy used in the Critical Age Periods Impacting the Trajectory of Obesogenic Lifestyles Project, which is an initiative based in NW Tasmania focused on assessing obesity prevention capacity. A social prescription model that facilitates the linkage of primary health screening with essential health care, education, and community resources through a dedicated “navigator” will be implemented. Four interlinked work packages will be implemented as part of the initial plan with each either building on existing resources or developing new initiatives.

**Results:**

A multimethod approach to triangulate insights from quantitative and qualitative research that enables the assessment of impact on individuals, community groups, and the health care system will be implemented within the initial pilot phase of the project.

**Conclusions:**

Literature is replete with rhetoric advocating complex system approaches to curtail obesity. However, real-life examples of whole-of-systems interventions operationalized in ways that generate relevant evidence or effective policies are rare. The diverse approach for primary prevention of obesity-related lifestyle diseases and strategies for improvement of health and well-being described in this instance will contribute toward closing this evidence gap.

**International Registered Report Identifier (IRRID):**

PRR1-10.2196/41280

## Introduction

Despite the absence of a widely accepted definition, social prescribing (SP) generally refers to the enablement of health care professionals to refer a patient or client to a social prescriber (eg, link worker, community connector, community navigator, health coach, health trainer, and so on). The social prescriber is responsible for the co-design of interventions to improve the individual’s health and well-being [1]. Once seldom acknowledged, or at best, paid “lip service,” social and behavioral determinants of health are now becoming increasingly more recognized as centrally critical to the provision of effective person-centered intervention in clinical practice and disease management [2]. Accordingly, a spate of SP-based interventions have been reported in recent times, including those from the United Kingdom and Americas [3], Scandinavia [4], and Australia [5,6]. Despite a paucity of definitive evidence regarding SP, benefits cited include improvements in health behaviors, mental health, and reductions in the use of tertiary care facilities for lifestyle diseases [1,7-10].

Obesity, one of the more prominent contemporary lifestyle-related diseases, has reached pandemic proportions globally and shows no signs of abating [11,12]. Consistent with global trends, the prevalence of obesity in Australia has increased in the past few decades, with 63.4% of adults and 27.6% of children with overweight and obesity in 2014-2015 [13]. If left unchecked, adult obesity and rates of severe obesity in Australia are predicted to reach 35% and 13%, respectively, by 2025 [14]. A further complication is that people living in geographically remote parts of Australia have a disproportionately higher prevalence of obesity. For instance, adults from regional (1.53 times higher) and rural areas (1.32 times higher) were more likely to have obesity than their counterparts from major city or urban areas [15]. Obesity also disproportionately affects low socioeconomic communities with a greater disease burden [16]. Tasmania, as a regional state with low socioeconomic status, has some of the highest rates of adult and childhood obesity and requires urgent intervention.

The rampant global prevalence of obesity is also an indication that the prevailing, disease-oriented biomedical approach to health service delivery is inadequate/ineffective at arresting the spread and mitigating the damaging consequences of the condition [17]. There is an urgent need to shift the focus from reactive disease-based treatments to incorporate some consideration of how individuals and communities are influenced by a growing global, regional, and local interdependence and an increasingly complex array of interlinking factors. Accordingly, more proactive, upstream, preventive efforts, and action to improve health and reduce obesogenic factors are required to arrest the increasing prevalence of obesity [18]. In essence, this requires a paradigm shift from the traditional “biomedical approach of care,” wherein the cause of illness is primarily thought to reside in the body, to a “biopsychosocial model,” which provides a basis for understanding the broader determinants of disease to arrive at treatment and service response plans [19].

Recent evidence from whole-of-community approaches anchored in systems science principles holds some promise in denting the harmful impact of obesity [20-22]. Dynamic systems theory posits that biological, social, and contextual factors interact in dynamic ways to influence health and participation across the lifespan [18]. Bronfenbrenner person-centered ecological framework allows the varied interactive interpersonal dynamics that influence development (and therefore health and obesity) to be examined [23]. At the center of the system, the individual brings their own unique characteristics, such as their age, genetics, life experiences, temperament, and coping strategies, and other familial, social, economic, and political layers of influence that sit around them [23]. Fundamentally, effective whole-of-system approaches are not limited by predefined agendas; instead, they are reliant on localized community input for context-specific, holistic, preventive work that acknowledges the synergistic interaction of a myriad of biological, social, economic, and environmental factors [24]. This approach has a commonality with effective SP interventions [25]. Following this logic, a community-driven SP approach could be just as effective in obesity prevention as any other initiative anchored in systems thinking. Herein, we discuss an SP protocol aimed at obesity prevention in Circular Head, a local government area (LGA) in Northwest (NW) Tasmania.

## Methods

### Ethical Considerations

Ethics approval was obtained for all procedures from the Human Research Ethics Committee (Tasmania) Network (H001864) and will be implemented in accordance with relevant guidelines and regulations stipulated in the Declaration of Helsinki. Both written and verbal consent (where practicable) will be obtained from all participants. This work was funded by a National Health and Medical Research Council (NHMRC) grant as part of the Critical Age Periods Impacting the Trajectory of Obesogenic Lifestyles (CAPITOL) Project. The study funder had no role in study design, collection, analysis, or interpretation of the data, in writing the report, or in the decision to submit the article for publication. The contents of this article are the responsibility of the authors and do not reflect the views of the NHMRC.

### Setting: CAPITOL Project NW Tasmania

The CAPITOL Project is a community-based “systems thinking” initiative based in the NW with an intentional focus on assessing obesity prevention capacity (OPC), engaging the community in developing OPC, and evaluating OPC development efforts. The central tenet of this work acknowledges the sustained multidisciplinary collaborations required to manage lifestyle risk factors, including nutrition, physical inactivity, smoking, and mental health, which can either be a cause or a consequence of excess adiposity. While NW Tasmania represents an expansive land mass, for reasons of practicality, the CAPITOL project operations are limited to 3 sentinel sites (Burnie, Circular Head, and Devonport). Sentinel sites are communities from which in-depth data can be collected, which has a high degree of transferability [26].

The current health profile of NW Tasmania, combined with its predominantly rural, geographically dispersed and socioeconomically disadvantaged population, is a hotbed for the proliferation of lifestyle-related disease. By virtue, the region also has a high demand/need for health promotion [27,28]. Nonetheless, the existing preventive health system in the region is underdeveloped and underresourced, which compounds existing health issues and stymies the progress of well-intentioned preventive efforts [29]. In addition to the lack of resourcing, the predominant narrative in the region has traditionally been a deficit-based view of health. To help circumvent these challenges, the holistic systems design of the CAPITOL Project is focused on asset-based community development alongside a primary prevention focus (ie, keeping healthy people healthy) with heightened attention on critical life stages (ie, mothers, infants, children, adolescents, and so on).

### Context: Circular Head

Consistent with other socioeconomically disadvantaged and remote areas, the Circular Head community displays an elevated prevalence of “risk factors” that contribute to adverse health and well-being outcomes. For instance, over 95% and 54% of the population does not meet the vegetable and fruit intake guidelines, respectively [30]. Further, one-fourth of the adult population do not take part in sufficient levels of moderate and vigorous physical activity (PA). Unfortunately, many of these risks are associated with the social determinants of health, including educational attainment, employment status, and housing conditions [31,32]. Low levels of health literacy, particularly in relation to food and PA, help to exacerbate the suboptimal health profile of the LGA (CAPITOL, unpublished data).

**Figure 1:**
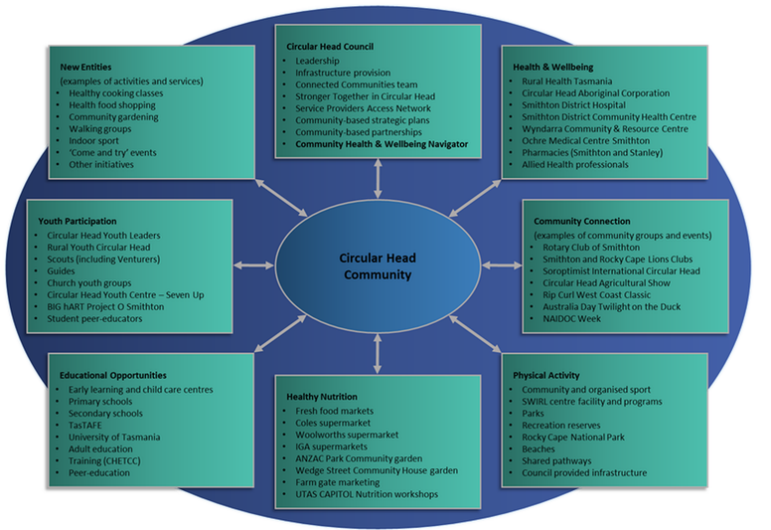
Assets and strengths of Circular Head.

Poor access to health professionals, including in allied health, medicine, nursing, and midwifery, is also endemic in the area [33]. Recent situational analyses revealed significant shortages of general practitioners, specialists, dietitians, exercise physiologists, and physiotherapists in Circular Head. Many health professionals are itinerant with the community, frequently enduring long wait periods for health care, extensive travel to access health care, and poor availability and access to preventative health care options (CAPITOL, unpublished data). Despite the availability of a range of community and health services (Figure 1), there is also a perceived lack of preventive services and health promotion interventions in the region. While explanations for the shortcomings cited are manifold, primary reasons include poorer community engagement, a declining volunteer base, and adverse climatic conditions (CAPITOL, unpublished data).

### Objectives

**Figure 2:**
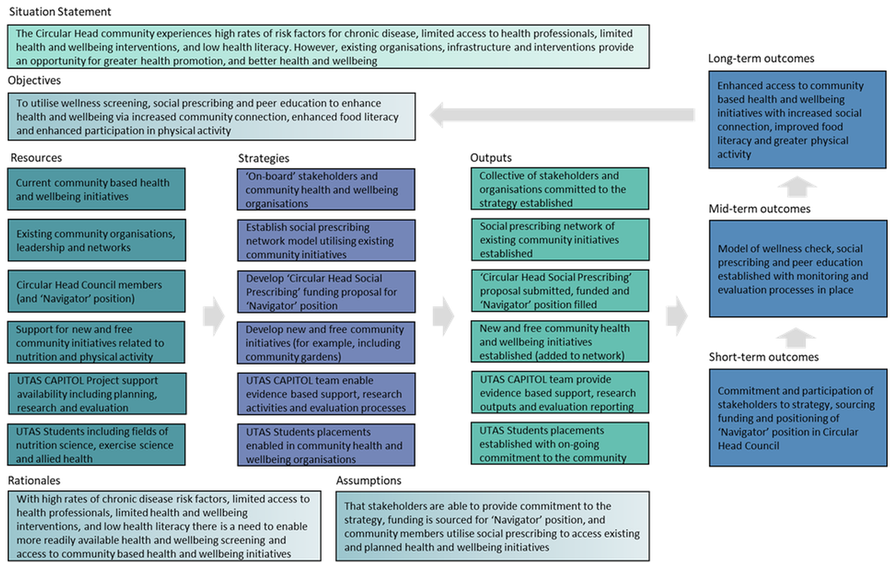
Circular Head social prescription logic model.

Despite the challenges, the Circular Head region has many significant assets that, if utilized optimally, may enable and help sustain community-driven change (Figure 1). To maximize success, there is an urgent need to link existing strategies, networks, committees, infrastructure, community-based interventions, organizations, and partnerships (Figure 2). The harnessing of existing capacity requires buy-in and leadership from local government and service organizations in the community.

The proposed SP intervention has the following three key objectives: (1) to build a sustainable program for Circular Head based on SP, peer education, and health screening to minimize the incidence of obesity and related lifestyle diseases; (2) to increase service and workforce connectivity and collaboration and initiate the introduction of new services and activities for obesity prevention and community health promotion; and (3) to enhance health and well-being and minimize preventable adverse health outcomes of obesity and related lifestyle diseases in the Circular Head LGA through enhancement of food literacy and enabling better nutrition, enhancement of physical literacy and increasing habitual personal activity levels, and improvement of mental health through increased community connectedness and reduction of social isolation.

### Strategy Mix

#### Theoretical Background

Historically, overcoming obesity has proven to be extremely challenging given the multifarious etiology of the condition, including individual and societal influences [34]. Prevention of contemporary lifestyle diseases, including obesity, requires multilevel, strategic, cross-sectoral, and systems-thinking solutions implemented in a practice environment supported by realist and capacity building approaches [35-41]. As such, all health promotion and the SP that derives from this research will be guided by realist principles, situated, and informed by evidence, and local context. The identification of context-mechanism-outcome configurations applicable to a variety of scenarios will be utilized to gather intel and explore causal mechanisms of the current lifestyle-related disease predicament in Circular Head.

#### Project Governance

The University of Tasmania (UTAS) will be the facilitating partner and legal entity responsible for this place-based project. The Circular Head Council will act as the intermediary organization along with key stakeholders to ensure place-based representation and insights. All procedures are informed by the specifications of the Human Research Ethics Committee (Tasmania) Network and will conform to the guidelines of the NHMRC’s National Statement on Ethical Conduct in Human Research 2007 (updated 2018). To this end, both written and informed consent will be obtained from the participants where practicable. A project steering group (ie, Circular Head Health and Well-being Coalition) will be formed with the imprimatur of key place-based partners and composed of members from the wider stakeholder group. Key stakeholders will be identified in a process of individual stakeholder consultation and stakeholder identification (through purposive sampling using publicly accessible contact information and professional networks) in line with the methodology of the formative stage of the CAPITOL Project. Specifically, key stakeholders will be individuals living in the sentinel site with attributes including high levels of interest, influence, expertise, or community connection as evidenced by occupational position/role, social standing in the community, or as nominated by other stakeholders consulted in previous community consultations. Each step of the governance process will be structured to ensure that the voice of key stakeholders informs or guides the research and the interpretation of findings across each work package. The Circular Head Council will be responsible for oversight of the co-design, development, and implementation of the project, with accountability for outcomes and impacts intended to be shared by all parties involved.

#### Project Implementation

##### Overview

**Figure 3:**
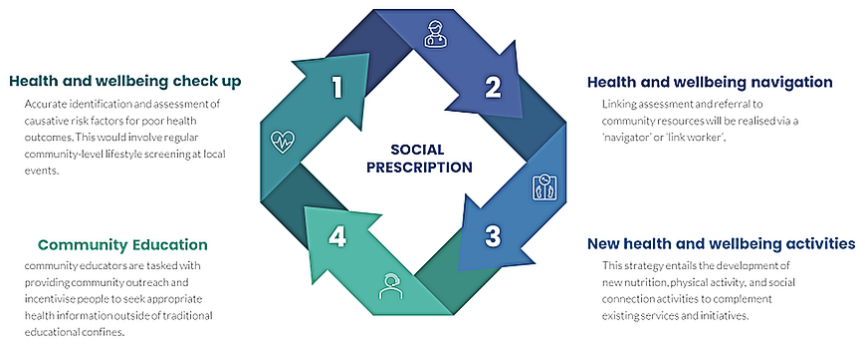
Social prescription model.

Overall, a social prescription model that facilitates the linkage of primary health screening with essential health care resources through a dedicated navigator will be implemented. Four (interlinked) main work packages will be implemented as part of the initial plan with each focus either building on existing resources or developing new initiatives (Figure 3). The opportunity to partake in this SP initiative will be open to all residents of the Circular Head LGA with recruitment exclusively based on convenience and snowballing. While the realist approach and the fluidity of study confines (as is typical for most SP interventions) renders precise a priori sample size calculations nonviable, where practicable, we intend to recruit as many participants as needed to achieve meaningful outcomes. For instance, the upper limit of the sample size for focus groups will be based on the attainment of “theoretical saturation.”

##### Health and Well-being “Checkup”

The primary aim of health and well-being “checkup” screening is to assist in the accurate identification and assessment of causative risk factors for poor health outcomes. This would involve regular community-level lifestyle screening at local events, workplaces, and sporting groups by qualified as well as trainee (eg, nutrition science, exercise science, nursing, and medical students) health care professionals. It is anticipated that these additional checkups will complement the extant visitations to health care professionals (eg, general practitioners, specialists, and allied health professionals), although they are not intended to replace any existing service. Overall, the checkups will initiate the SP referral process that will primarily be managed by a dedicated navigator.

##### Health and Well-being “Navigation”

Linking assessment and referral to community resources will be realized via a navigator or “link worker.” As evidenced in literature to date, SP protocols in the past have utilized a wide array of (mostly) health workers for the navigation role [6]. Responsibilities of the link worker can be manifold and may include coproducing health goals and action plans, enabling of resident-health service and activity interaction, and providing feedback at multiple stakeholder levels. Existing evidence indicates that such roles have been particularly efficacious in disadvantaged communities where more intensive support is inherently required due to accentuated complexity of health challenges [42]. In this study, the navigator would be employed by the Circular Head LGA and act as the conduit between residents, health care services, and preventive activities.

##### Community “Education”

Peer education is the process of learning and sharing information with peers in the community and is a key strategic component to amplify health and well-being messaging. In this proposed work model, community educators are tasked with providing community outreach and incentivizing people to seek appropriate health information outside of traditional educational confines. Upskilling community volunteers and leaders would provide a source of community-based support and education for other components of the strategy. Specifically, a smorgasbord of group-based education sessions cofacilitated by project staff, generation of culturally and contextually appropriate education material, and the provision of appropriate technologies to provide information on nutrition and PA will be of central focus within this package. Working examples will be drawn from existing programs such as the Family Food Patch program which uses peer education to help improve health and well-being of Tasmanian children and families through promotion of healthy eating and PA.

##### New Health and Well-being Activities

**Figure 4:**
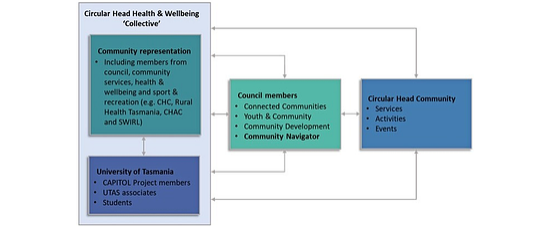
Organizational structure of the Health and Well-being Coalition.

This strategy entails the development of new nutrition, PA, and social connection activities to complement existing services and initiatives. A key component of this work model will include the establishment of the Circular Head Health and Well-being Coalition (Figure 4), which will provide guidance and direction for all co-designed health promotion activities within the SP framework. The basis of the coalition is entrenched in the legacy partnerships between the CAPITOL Project and the Circular Head Council and will be emboldened by further collaboration with community and service organizations, including Rural Health Tasmania, Circular Head Aboriginal Corporation, and the Smithton Well-being Indoor Recreation and Leisure center. While it is premature to confirm precise details about the potential activities in this SP initiative, extant literature highlights examples of multifarious “prescribing schemes” including arts, books, education, and exercise [43].

## Results

**Figure 5:**
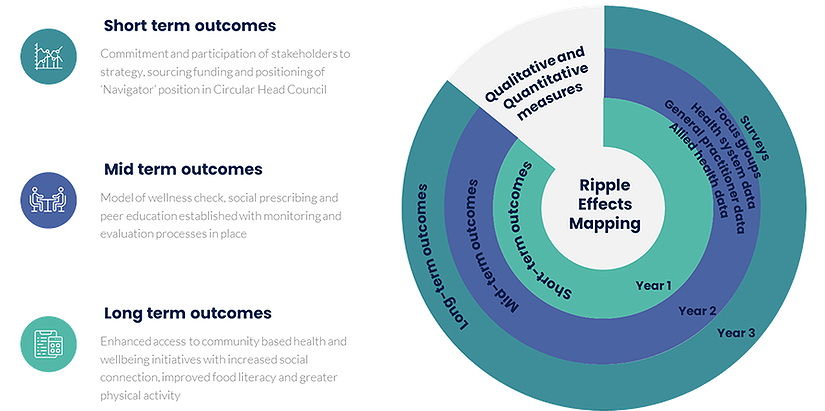
Summary of outcome and evaluation strategies. Notwithstanding the complexities of simultaneous measurement of numerous interlinked components of the systemwide SP initiative, a robust set of evaluative frameworks will be applied to all levels (development, formative and summative) and activities.

It is imperative that SP models undergo rigorous evaluation using systems science approaches and mixed methods research to ensure understanding of what worked, why it worked, and in what contexts. A multimethod approach to triangulate insights from quantitative and qualitative research that enables the assessment of impact on individuals, community groups, and the health care system will be implemented within the initial 3-year pilot phase of the project. For instance, a range of health and well-being and health care usage outcomes will be measured (Figure 5) to evaluate the efficacy of the SP program. Evaluation will include a review of implementation efforts, project impacts, plans for sustainability, and feedback from participants, educators, administrators, and stakeholders.

Given traditional evaluation tools are often ill-suited for co-design work in community settings, we intend to use a ripple effects mapping approach, a participatory technique to engage stakeholders in visually mapping project efforts and results to collect impact data [44]. Specifically, focus group techniques to work with participants to process and reflect on their lived experiences and collectively and visually map project efforts and results. We envisage that the use of mapping will trigger verbal responses, increase the frequency and depth of responses, and aid participants in recalling, organizing, and reflecting on experiences. It is intended that a series of modified nominal group technique (mNGT) consensus method group processes will be executed annually to evaluate progress. The core steps (silent generation, round robin, clarification, and voting [ranking and rating]) [45] of the mNGT approach will facilitate collaborative decision-making and, importantly, enhance equitable participation of stakeholders. Where possible, the progress of key components will also be compared against the 5 pillars (a common agenda, shared measurement systems, mutually reinforcing activities, continuous communication, and backbone support organizations) of the “collective impact” framework [46].

Exact details of the outcome measures will be decided in consultation with the community, as their suitability will be based on nature of referrals, prescribed activities, participant needs, and resourcing. Nevertheless, in line with the popular approaches in contemporary SP initiatives, it is envisaged that a mix of subjective well-being, quality of life, behavior change, physiological changes, frequency of health service access, medication usage, and development of social networks will be measured either quantitatively or qualitatively [43,47]. In addition, where practicable, the social return on investment will also be measured using contextually appropriate inputs [48,49].

## Discussion

This proposal describes a diverse approach for primary prevention of obesity-related lifestyle diseases and strategy for improvement of future health and well-being in a remote and socioeconomically disadvantaged population. The SP approach anchored in systems thinking described above could be an effective means of moderating prevalence and consequences of obesity at a community level. Key focal points of this intervention include peer education, health screening, service accessibility, workforce connectivity, food literacy, better nutrition, physical literacy, habitual PA, mental health, social isolation, and community connectedness. The Circular Head Health and Well-being Coalition will be established to address health and well-being inequities affecting people in the region. This collective will be responsible for providing direction for the implementation of the whole-of-community Circular Head Health and Well-being Strategy, broadly based on principles of SP.

As per the global accord in contemporary times, Australia also has a health care system that has a treatment-oriented and reactive bias. For instance, the extant 31 Primary Health Networks of Australia that prioritize mental health, First Nation people’s health, population health, health workforce, digital health, aged care, and alcohol/drugs are fundamentally designed for “commissioning of health services” for consumers with diagnosed conditions. Further, Australia is one of the lowest spenders on preventive health (~2 billion per annum; ~1.5% of the total health budget; ~US $89 per person every year) among the Organisation for Economic Co-operation and Development nations such as Canada, the United States, and New Zealand. This is despite public health research consistently indicating that most preventive health interventions are cost-effective (mainly through cost reductions in treating complex conditions), improve quality of life and longevity, and minimize the burden on the health care system as a whole [50]. Therefore, considering the urgency of obesity prevention and health intervention in Circular Head, the SP approach we describe is both timely and necessary.

The effectiveness of an SP initiative may also rely on the collective buy-in from the wider stakeholder group, including, most importantly, the community members. As such, consistent and frequent education regarding the nature and benefits of the program from inception to conclusion will be vital. Further, expansive networking and development of governance structures that entail strong leadership from all stakeholder groups is also of utmost importance. The whole-of-systems approach proposed in this research with SP as the central tenet is expected to address all of the above and improve community cohesion, social engagement, reciprocity in peer support, and community confidence in accessing pertinent resources for improving health and well-being outcomes. Community ownership of public health initiatives has shown significant promise in other settings [51-54], with a satisfactory compromise between community lived experience and contemporary scientific evidence being the key driver of success.

Translation of lifestyle disease–related research findings into sustainable practice is a public health priority. However, existing research indicates that this transition is largely ineffective due to limitations such as insufficient scaling-up for systemwide integration and lack of emphasis on the nonlinear and complex overlapping of various (development, implementation, sustainment, etc) program stages [55-57]. Much of the existing evidence base being underpinned by linear models of cause and effect is also exacerbating this issue. The whole-of-systems approach of the CAPITOL Project, along with 3 years of preliminary work [58-60] and early and frequent engagement of stakeholders in all aspects of project planning, is expected to elicit maximal translatory potential of the current SP initiative. Further, community-wide, representative consultation and equity in decision-making and agenda setting is also considered best practice in this line of intervention [53].

This proposed SP approach can have several limitations. Despite the potential benefits, a robust and generalizable protocol for satisfactory data collection and evaluation of any SP protocol will be challenging. For instance, given the inherent heterogeneity of designs in SP initiatives [61], it is not feasible to gain a wider consensus on the most appropriate outcome measures [62]. Further, nonlinearity of SP initiatives and the inevitability of multiple, fleeting, and intersecting components can also exacerbate this complexity [63]. Consequently, we posit that a realist approach, which acknowledges a set of context-specific outcomes (both biomedical and social determinants) of importance, will yield the most effective SP system in any given setting. As the outcomes will be reliant on local contextual factors, the generalizability of the findings will also be limited and might not be particularly applicable outside of the area under study. Further, any number of commonly reported practical challenges to generating evidence around SP, such as forging collaborative relationships while maintaining researcher independence, lack of a control group for outcome comparison, accuracy of health and social care service usage data, sustained resourcing, and consent and privacy matters, can also be relevant in this instance [64].

The need for complex processes and outcomes that drive change within a whole system and the necessity of doing away with linear approaches to mitigating complex public health issues has been echoed consistently in recent times [65]. Nevertheless, the practicalities of real-life implementation of whole-of systems approaches are ill-defined. This proposal describes a practicable set of approaches for stakeholder engagement and community participation in uplifting health and well-being standards in a locality with poor socioeconomic and health outcomes.

The Tasmanian Charter of Health Rights and Responsibilities states the following:

When viewed as a partnership, the relationship between the health service consumer and the health service provider is more likely to benefit the health outcomes of the service consumer. While the health service provider has a responsibility to meet certain rights of the health service consumer, the consumer in turn, should also assume some responsibility for their own health care.

The proposed SP initiative will provide a viable opportunity to the residents in Circular Head to have a real-life, at scale attempt to co-design a sustainable solution to their health and well-being challenges while inculcating democratic partnerships between all project members as collaborators through every stage of the project. Further, the proposed program will help to increase access to peer and other support workers able to assist community members to navigate the system and receive adequate, timely, and situational support—a need frequently highlighted in the region [66].

## Abbreviations

CAPITOL: Critical Age Periods Impacting the Trajectory of Obesogenic Lifestyles
LGA: local government area
mNGT: modified nominal group technique
NHMRC: National Health and Medical Research Council
NW: Northwest
PA: physical activity
OPC: obesity prevention capacity
SP: social prescribing

## Data Availability

The data sets generated during or analyzed during the current study will be available from the corresponding author on reasonable request.
